# The effects of economic status on metabolic control in type 2 diabetes mellitus at 10 metabolic management centers in China

**DOI:** 10.1111/1753-0407.13466

**Published:** 2023-09-05

**Authors:** Liping Gu, Yuhang Ma, Qidong Zheng, Weiqiong Gu, Tingyu Ke, Li Li, Dong Zhao, Yuancheng Dai, Qijuan Dong, Bangqun Ji, Fengmei Xu, Juan Shi, Ying Peng, Yifei Zhang, Tingting Shen, Rui Du, Jiaying Yang, Mei Kang, Yongde Peng, Yufan Wang, Weiqing Wang

**Affiliations:** ^1^ Department of Endocrinology and Metabolism, Shanghai General Hospital Shanghai Jiao Tong University School of Medicine Shanghai China; ^2^ Department of Internal medicine The Second People's Hospital of Yuhuan Yuhuan China; ^3^ Department of Endocrine and Metabolic Diseases, Shanghai Institute of Endocrine and Metabolic Diseases, Ruijin Hospital Shanghai Jiao Tong University School of Medicine Shanghai China; ^4^ Shanghai National Clinical Research Center for metabolic Diseases, Key Laboratory for Endocrine and Metabolic Diseases of the National Health Commission of the PR China, Shanghai Key Laboratory for Endocrine Tumor, State Key Laboratory of Medical Genomics, Ruijin Hospital Shanghai Jiao Tong University School of Medicine Shanghai China; ^5^ Department of Endocrinology The Second Affiliated Hospital of Kunming Medical University Kunming China; ^6^ Department of Endocrinology The First Affiliated Hospital of Ningbo University Ningbo China; ^7^ Center for Endocrine Metabolism and Immune Diseases, Beijing Luhe Hospital Capital Medical University Beijing China; ^8^ Department of Internal medicine of traditional Chinese medicine Sheyang Diabetes Hospital Yancheng China; ^9^ Department of Endocrinology and Metabolism People's Hospital of Zhengzhou Affiliated Henan University of Chinese Medicine Zhengzhou China; ^10^ Department of Endocrinology Xingyi People's Hospital Xingyi China; ^11^ Department of Endocrinology and Metabolism, Hebi Coal (group). LTD General hospital Hebi China; ^12^ Clinical Research Center, Shanghai General Hospital Shanghai Jiao Tong University School of Medicine Shanghai China

**Keywords:** glycosylated hemoglobin, gross domestic product per capita, healthy lifestyle, metabolic management Centre, type 2 diabetes mellitus

## Abstract

**Objective:**

This study investigated the association of economic status with metabolic index control in type 2 diabetes mellitus (T2DM) patients.

**Methods:**

In total, 37 454 T2DM patients from 10 National Metabolic Management Centers in China were recruited and categorized into two groups: a high‐gross domestic product (GDP) group (*n* = 23 993) and a low‐GDP group (*n* = 13 461). Sociodemographic characteristics, medical histories, and lifestyle factors were recorded. Logistic regression and interaction analysis were performed to evaluate the association of economic status and healthy lifestyle with metabolic control.

**Results:**

Compared to the low‐GDP group, there were fewer patients with glycated hemoglobin (HbA1c) levels ≥7% in the high‐GDP group. Fewer patients with a high GDP had an abnormal metabolic state (HbA1c ≥ 7%, blood pressure [BP] ≥130/80 mm Hg, total cholesterol [TCH] ≥4.5 mmol/L or body mass index [BMI] ≥24 kg/m^2^). The risks of developing HbA1c ≥ 7% (odds ratios [OR] = 0.545 [95% CI: 0.515–0.577], *p* < .001), BP ≥ 130/80 mm Hg (OR = 0.808 [95% CI: 0.770–0.849], *p* < .001), BMI ≥ 24 kg/m^2^ (OR = 0.840 [95% CI: 0.799–0.884], *p* < .001), and an abnormal metabolic state (OR = 0.533 [95% CI: 0.444–0.636], *p* < .001) were significantly lower in the high‐GDP group even after adjustment for confounding factors. Younger participants; those with a family history of diabetes, normal weight, and a physical activity level up to standard; and those who did not drink alcohol in the high‐GDP group were predisposed to better glycemic levels.

**Conclusions:**

T2DM patients in economically developed regions had better metabolic control, especially glycemic control. A healthy lifestyle had an additive effect on achieving glycemic goals, even among high‐GDP patients.

## Graphical abstract

Highlights

- The risk of developing an abnormal metabolic state in type 2 diabetes mellitus patients with a higher gross domestic product per capita was significantly lower after adjustment for possible confounders.
- A healthy lifestyle had an additive effect on achieving glycemic goals, even in individuals with a better economic status.

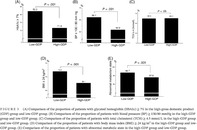

## 1 INTRODUCTION

The prevalence of diabetes has increased globally. China has the highest prevalence of diabetes, which increased from 0.76% in 1980 to 12.8% in 2017.^1^ With rapid economic growth in the past 40 years, there has been a strong disparity in regional development and personal income in China. Type 2 diabetes mellitus (T2DM) can be influenced by socioeconomic status at both the societal and individual levels. The gross domestic product (GDP) per capita of a city reflects the city's economic output per person and was calculated by dividing the GDP of the city by its population. GDP per capita is a global measure for gauging the prosperity of cities in regard to economic growth.

The prevalence of T2DM was positively related to socioeconomic status (indexed by average family income). After controlling for potential confounding factors, people in higher socioeconomic status groups were more likely to have T2DM.^2^, ^3^, ^4^ Previous studies have also found that a 1% higher GDP in the service industry was associated with a 1% higher diabetes prevalence (OR: 1.01 [95% CI: 0.99–1.02]).^5^ Onyango found that the increase in DM prevalence was accompanied over the same period by a concomitant rise in GDP per capita and physical inactivity (as measured by increased urbanization and declining proportion of agricultural and forestry wage jobs) in Kenya.^6^

With the increase in diabetes prevalence in China, the metabolic control status of diabetic patients seems to be serious. The proportion of patients with glycated hemogloblin (HbA1c) <7% was 47.7%, and the proportion of patients achieving 3B goals (control of blood glucose, blood pressure [BP], and blood lipids) was only 5.6% in the Nationwide Assessment of CVD [Cardiovascular Disease] Risk Factors: Blood Pressure, Blood Lipid, and Blood Glucose, in Chinese Patients with Type 2 Diabetes (T2D)–China Cardiometabolic Registries (CCMR‐3B) study.^7^ To meet the great challenges and heavy workloads of diabetes management in China, metabolic management centers (MMCs) were launched. A detailed introduction of the MMC program can be found in previous publications (ClinicalTrials.gov number, NCT03811470).^8^, ^9^

Previous studies have focused mainly on the relationship between socioeconomic conditions and the prevalence of T2DM. However, few studies have investigated the economic status and metabolic control status in T2DM patients. Therefore, the aim of the present study was to examine the relationship between the metabolic index control rate and GDP per capita among T2DM patients from 10 MMCs. An interaction analysis was further performed in subgroups stratified by clinical characteristics, lifestyle behaviors and medical histories.

## 2 METHODS

### 2.1 Study population and design

**FIGURE 1:**
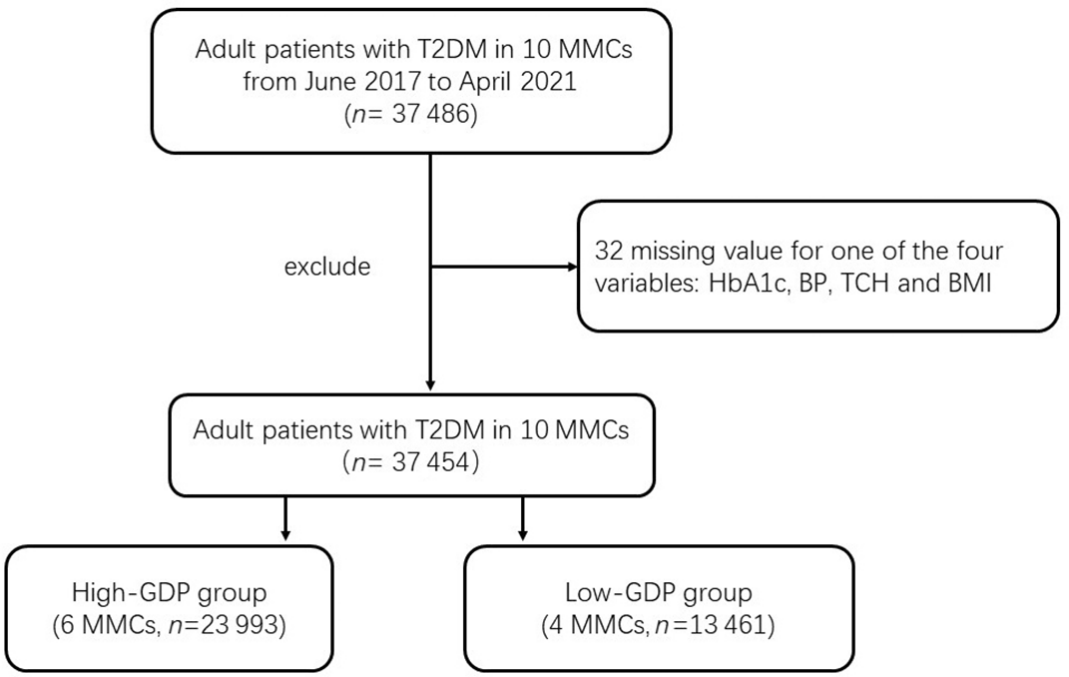
Study population. BMI, body mass index; BP, blood pressure; GDP, gross domestic product; HbA1c, glycated hemoglobin; MMC, metabolic management center; T2DM, type 2 diabetes mellitus; TCH, total cholesterol.

**FIGURE 2:**
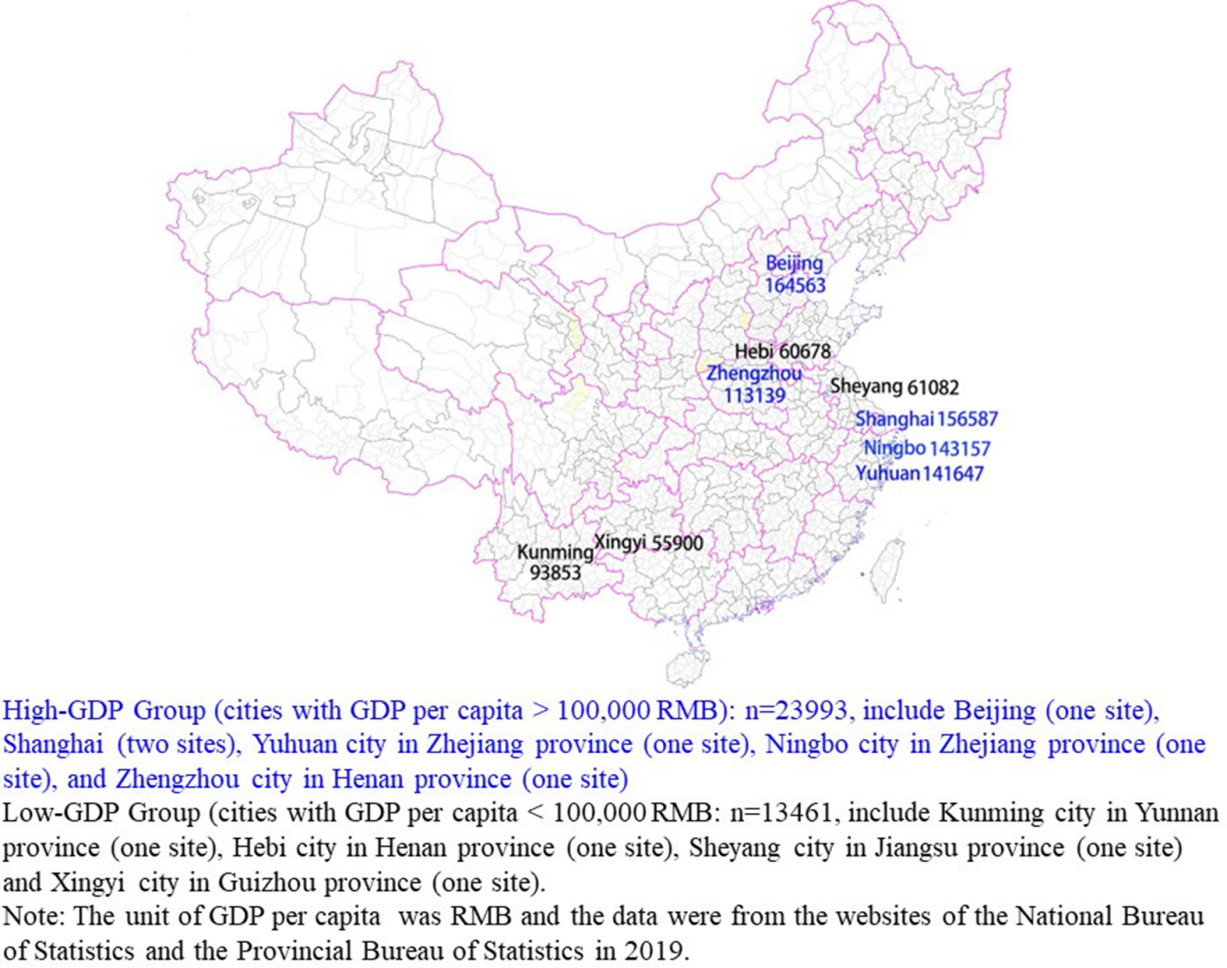
Per capita gross domestic product (GDP) and grouping diagram of cities where the 10 metabolic management centers (MMCs) were located.

We used data from 10 national MMCs distributed in nine cities and seven provinces in China. These MMCs, with good quantity and quality of diabetes patient management, were all located in cities that are representative of cities' GDP per capita in China. From June 2017 to April 2021, 37 486 adult patients with T2DM from the 10 sites participated in a comprehensive baseline investigation, which included a standardized questionnaire, anthropometric measurements, and laboratory tests. The protocol of this project was published previously.^10^, ^11^, ^12^, ^13^ Adult T2DM patients who were willing to accept MMC management and follow‐up gained entry into the MMCs. In our study, as long as there was no missing value for any of the four variables (HbA1c, BP, total cholesterol [TCH], and body mass index [BMI]) the subject was included. Therefore, a total of 37 454 participants from the 10 hospitals were enrolled and categorized into a high‐GDP group (average per capita GDP: 143819 RMB [renminbi]) and a low‐GDP group (average per capita GDP: 67878 RMB) according to the average per capita GDP (100 000 RMB) of the cities where the 10 centers are located (Figures 1 and 2). The GDP data were from the websites of the National Bureau of Statistics and the Provincial Bureau of Statistics in 2019. The high‐GDP group (*n* = 23 993) included six centers, and the low‐GDP group (*n* = 13 461) included four centers.

The study protocol was approved by the Ethics Review Committee of Ruijin Hospital and the other participating centers. The study was performed in accordance with the Declaration of Helsinki, and informed consent was obtained from each participant.

### 2.2 Data collection

The data were collected in local MMCs by trained staff according to a standard protocol.^8^ Sociodemographic characteristics, medical history, and lifestyle factors were recorded according to a standard operation procedure.^10^, ^13^ Briefly, height and body weight were measured, and BMI was calculated as the weight in kilograms divided by the height in meters squared. Waist circumference was measured on standing participants midway between the lower edge of the costal arch and the upper edge of the iliac crest. The participants underwent a standard steamed bread meal test after an overnight fast, and blood samples were collected at fasting and 2 h postprandial during the test. Fasting blood glucose (FBG), 2‐h postprandial blood glucose (PBG), fasting serum C peptide, postprandial serum C peptide, HbA1c, TCH, triglycerides (TGs), low‐density lipoprotein cholesterol (LDL‐C), and high‐density lipoprotein cholesterol (HDL‐C) were tested in local MMCs. Homeostasis model assessment of insulin resistance (HOMA2‐IR) and HOMA‐2β were calculated based on FBG and fasting C peptide concentrations (University of Oxford, Oxford, UK).^14^

### 2.3 Definition of variables

Diabetes was diagnosed if an individual had an FBG ≥ 7.0 mmol/L, PBG ≥11.1 mmol/L, or self‐reported previous diagnosis by physicians in accordance with the 1999 World Health Organization criteria.^15^ T2DM was confirmed by a qualified physician at each site. The international physical activity questionnaire was used to assess each patient's level of physical activity.^16^ The standard metabolic control targets were revised slightly according to the guidelines of diabetes prevention and treatment in China.^17^ An abnormal metabolic state was defined as HbA1c ≥ 7%, BP ≥130/80 mm Hg, TCH ≥4.5 mmol/L, or BMI ≥24 kg/m^2^. A physical activity level up to standard was defined as meeting the following conditions: moderate intensity exercise ≥150 min/week, vigorous exercise ≥75 min/week, or moderate intensity + vigorous exercise ≥150 min/week. Alcohol consumption habits were classified as current drinking or not. Current drinking referred to drinking every week or almost every week. Ideal smoking was defined as never smoking or quitting smoking for >12 months.

### 2.4 Statistical analyses

Statistical analyses were performed with R software, version 4.0.5. The data are expressed as the means ± SDs or median (interquartile range) for continuous variables and count (percentage) for categorical variables. Data were tested for normality and logarithmically transformed for statistical analysis when needed. To compare the differences between two groups, a *t* test was used for continuous variables, and the chi‐square test was used for categorical variables. Logistic regression models were used to calculate odds ratios (ORs) and 95% confidence intervals (CIs) for the ratio of HbA1c ≥ 7%, BP ≥130/80 mm Hg, BMI ≥24 kg/m^2^, TCH ≥4.5 mmol/L, and abnormal metabolic state according to different GDP groups. In the analysis of abnormal metabolic state, participants (*n* = 36 264) with no missing HbA1c, BP, BMI, or TCH data were included. In a further analysis, *p* for interaction analysis was used in the stratified subgroup analysis of HbA1c control. Logistic regression models and *p* for interaction analysis were all performed after adjustment for the following potential confounders: sex, age, diabetes duration, family history, history of hypertension, history of hyperlipidemia, BMI, physical activity, alcohol consumption habits, and smoking habits. All *p* values were two tailed, and a *p* value <.05 was considered statistically significant.

## 3 RESULTS

### 3.1 General characteristics and metabolic parameters of subjects with type 2 diabetes in different GDP groups

Compared to the low‐GDP group, patients in the high‐GDP group seemed to be younger (*p* < .001) and were more likely to be male (male, 60.8% vs. 52.5%, *p* < .001). Regarding medical history, patients in the high‐GDP group had a lower proportion of history of hypertension and shorter diabetes duration (*p* < .05). In addition, their family history of DM and history of dyslipidemia were significantly higher (*p* < .001). However, there were no significant differences in BMI or waist–hip ratio between the two groups.

Compared to the low‐GDP group, the systolic BP, diastolic BP, FPG, PPG, and HbA1c levels of the high‐GDP groups were lower (*p* < .001). The HOMA2‐β was higher and the HOMA2‐IR was lower among subjects in the high‐GDP group than among those in the low‐GDP group. Regarding the lipid profile, serum TGs and HDL‐C were lower in subjects with high GDP than in those with low GDP (*p* < .001). However, there were no significant differences in serum TC and LDL‐C between the two groups. All analyses are shown in Table 1.

**TABLE 1:** Demographic and metabolic characteristics of subjects with type 2 diabetes stratified by different gross domestic product (GDP) group.

Variable	Total	Low‐GDP	High‐GDP	*p* value
N	37454	13461	23993	
$\mathrm{Age}\;\left(\text{years}\right)$	54.3±11.5	55.9±10.3	53.4±12.0	<.001
$\text{Gender}\;\left(\text{male},\%\right)$	57.8	52.5	60.8	<.001
$\mathrm{DM}\;\text{family history}\;\left(\%\right)$	51.9	45.9	55.4	<.001
$\text{History of hypertension}\;\left(\%\right)$	42.1	44.0	41.0	<.001
$\text{History of dyslipidemia}\;\left(\%\right)$	29.8	27.7	31.1	<.001
$\text{Diabetic duration}\;\left(\text{years}\right)$	6.9±6.9	7.5±6.7	6.6±7.0	<.001
$\text{Drinking}\;\left(\mathrm{yes},\%\right)$	11.5	7.6	13.7	<.001
$\text{Ideal}-\text{smoking}\;\left(\mathrm{yes},\%\right)$	73.6%	76.0%	72.2%	<.001
$\text{Physical activity}\;\mathrm{up}\;\text{to standard}\;\left(\%\right)$	10.10	10.35	9.96	.239
$\text{Family income}\;\left(>100\;000\;\mathrm{RMB}/\text{year},\%\right)$	36.1	16.0	51.3	<.001
BMI(kg/m ^2^ )	26.00±3.86	26.05±3.69	25.98±3.95	.082
WHR	0.94±0.06	0.94±0.06	0.94±0.06	.981
$\mathrm{SBP}\;\left(\mathrm{mm}\;\mathrm{Hg}\right)$	132.4±18.8	134.2±20.3	131.3±17.8	<.001
$\mathrm{DBP}\;\left(\mathrm{mm}\;\mathrm{Hg}\right)$	77.8±11.6	78.5±11.9	77.3±11.3	<.001
$\mathrm{FBG}\;\left(\text{mmol}/L\right)$	9.47±3.83	10.11±4.29	9.12±3.50	<.001
$\mathrm{PBG}\;\left(\text{mmol}/L\right)$	15.94±5.41	17.64±5.44	14.97±5.16	<.001
$\mathrm{HbA}1c\;\left(\%\right)$	8.71±2.16	9.13±2.22	8.46±2.09	<.001
$\text{Fasting}\;C-\text{peptide}\;\left(\mathrm{\mu g}/L\right)$	$2.04\;\left(1.40,2.84\right)$	$2.16\;\left(1.39,3.05\right)$	$2.00\;\left(1.40,2.73\right)$	<.001
$2\;h\;C-\text{peptide}\;\left(\mathrm{\mu g}/L\right)$	$4.84\;\left(3.15,7.00\right)$	$4.94\;\left(3.17,7.17\right)$	$4.79\;\left(3.13,6.90\right)$	.012
HOMA2−IR	$1.9\;\left(1.3,2.7\right)$	$2.10\left(1.4,3.0\right)$	$1.8\;\left(1.3,2.5\right)$	<.001
$\text{HOMA}2-\beta \;\left(\%\right)$	$48.1\;\left(29.3,74.1\right)$	$45.2\;\left(26.9,73.1\right)$	$49.6\;\left(30.8,74.5\right)$	<.001
$\mathrm{TG}\;\left(\text{mmol}/L\right)$	$1.61\;\left(1.11,2.45\right)$	$1.75\;\left(1.19,2.69\right)$	$1.54\;\left(1.07,2.32\right)$	<.001
$\mathrm{TC}\;\left(\text{mmol}/L\right)$	4.97±1.33	4.97±1.35	4.98±1.31	.520
$\mathrm{HDL}-C\;\left(\text{mmol}/L\right)$	1.19±0.34	1.24±0.37	1.16±0.31	<.001
$\mathrm{LDL}-C\;\left(\text{mmol}/L\right)$	2.99±1.00	3.00±1.03	2.98±0.98	.091

*Note*: High‐GDP group: T2DM in cities with GDP per capita >100 000 RMB, Low‐GDP group: T2DM in cities with GDP per capita <100 000 RMB. Pairwise comparisons, High‐GDP group vs. Low‐GDP group.

Abbreviations: BMI, body mass index; DBP, diastolic blood pressure; FBG, fasting blood glucose; HDL‐C, high‐density lipoprotein cholesterol; HOMA2‐IR, homoeostasis model assessment of insulin resistance; HOMA2‐β, homoeostasis model assessment of β cell function; LDL‐C, low‐density lipoprotein cholesterol. M, male; PBG, postprandial blood glucose; RMB, renminbi; TC, total cholesterol; TG, triglyceride; SBP, systolic blood pressure; WHR, waist–hip ratio.

### 3.2 There were fewer patients with HbA1c ≥ 7%, BP ≥130/80 mm Hg, BMI ≥24 kg/m^2^, and abnormal metabolism in high‐GDP regions

**FIGURE 3:**
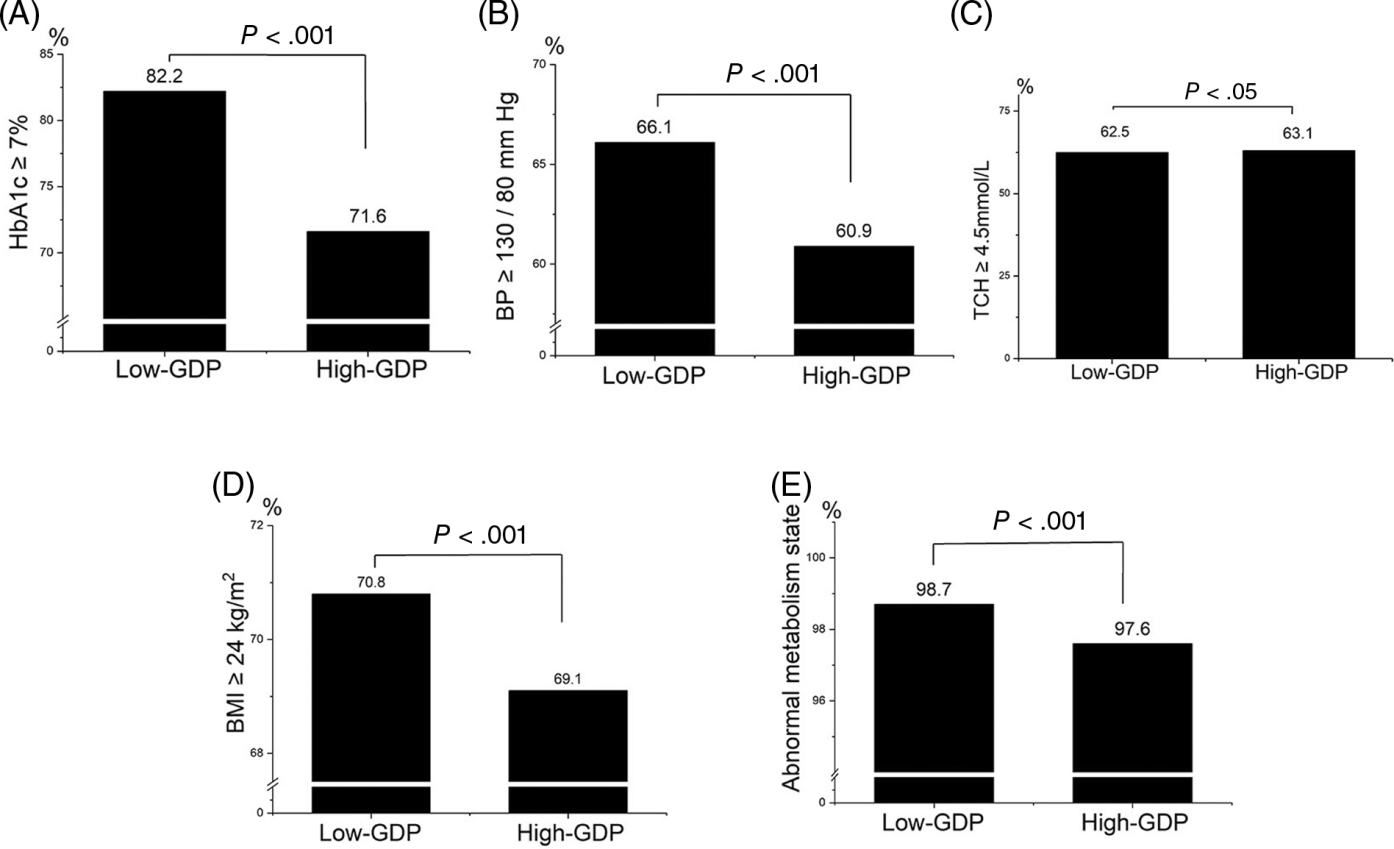
(A) Comparison of the proportion of patients with glycated hemoglobin (HbA1c) ≥ 7% in the high‐gross domestic product (GDP) group and low‐GDP group. (B) Comparison of the proportion of patients with blood pressure (BP) ≥ 130/80 mmHg in the high‐GDP group and low‐GDP group. (C) Comparison of the proportion of patients with total cholesterol (TCH) ≥ 4.5 mmol/L in the high‐GDP group and low‐GDP group. (D) Comparison of the proportion of patients with body mass index (BMI) ≥ 24 kg/m^2^ in the high‐GDP group and low‐GDP group. (E) Comparison of the proportion of patients with abnormal metabolic state in the high‐GDP group and low‐GDP group.

As shown in Figure 3, the percentage of patients with HbA1c ≥7% in the high‐GDP group was 71.6%, whereas it was 82.2% in the low‐GDP group (*p* < .001). Additionally, compared to the low‐GDP group, the proportion of patients with BP ≥130/80 mm Hg or BMI ≥24 kg/m^2^ in the high‐GDP group was much lower (60.9% vs. 66% and 69.1% vs. 70.8%, respectively, *p* < .001). Regarding the abnormal metabolic state (HbA1c ≥ 7%, BP ≥130/80 mm Hg, TCH ≥4.5 mmol/L, or BMI ≥24 kg/m^2^), the proportion of patients in the high‐GDP group was 97.6%, which was lower than that in the low‐GDP group (98.7%, *p* < .001). There were no significant differences in the proportion of patients with TCH ≥4.5 mmol/L between the two groups.

### 3.3 After adjusting for confounding factors, the risk of HbA1c ≥ 7%, BP ≥130/80 mm Hg, BMI ≥24 kg/m^2^, and abnormal metabolic status was significantly lower among patients with higher GDP per capita

Compared with the group with low GDP, the risk of HbA1c ≥ 7%, BP ≥130/80 mm Hg, BMI ≥24 kg/m^2^, and abnormal metabolic state (HbA1c ≥ 7%, BP ≥130/80 mm Hg, TCH ≥4.5 mmol/L, or BMI ≥24 kg/m^2^) was lower by 47.3% (OR = 0.544 [95% CI: 0.516–0.574]), 18.2% (OR = 0.7999 [95% CI: 0.765–0.835]), 11.6% (OR = 0.925 [95% CI: 0.883–0.968]) and 44.8% (OR = 0.562 [95% CI: 0.472–0.665]), respectively, adjusted for sex and age.

When we further adjusted for potential influencing factors, such as diabetes duration, family history of DM, history of hypertension, history of hyperlipidemia, BMI, physical activity, alcohol consumption habits, and smoking habits, we found that the lower risk of metabolic abnormalities persisted in the high‐GDP group. Compared with low GDP, high GDP was associated with 45.5% lower odds of having an HbA1c ≥7% (OR = 0.545 [95% CI: 0.515–0.577]), with 19.2% lower odds of havingBP ≥ 130/80 mm Hg (OR = 0.808 [95% CI: 0.770–0.849]), with 16.0% lower odds of having an BMI ≥ 24 kg/m^2^ (OR = 0.840 [95% CI: 0.799–0.884]), and also with 46.7% lower odds of having a comprehensive risk of poor metabolic indexes (HbA1c ≥ 7%, BP ≥ 130/80 mm Hg, TCH ≥4.5 mmol/L, or BMI ≥24 kg/m^2^, OR = 0.533 [95% CI: 0.444–0.636]). With regard to TCH levels, there was no regional variation (OR = 0.992 [95% CI: 0.946–1.039]). All analyses are shown in Table 2.

**TABLE 2:** Odds ratios (95% CI) for metabolic indexes in subgroups analysis of HbA1c control.

Variables	Group	Model 1		Model 2		Model 3	
		OR (95% CI)	*p* value	OR (95% CI)	*p* value	OR (95% CI)	*p* value
HbA1c ≥ 7%	Low‐GDP	1	< .001	1	< .001	1	< .001
	High‐GDP	0.544 (0.516–0.574)		0.527 (0.499–0.556)		0.545 (0.515–0.577)	
BP ≥ 130/80 mm Hg	Low‐GDP	1	< .001	1	< .001	1	< .001
	High‐GDP	0.799 (0.765–0.835)		0.818 (0.782–0.855)		0.808 (0.770–0.849)	
TCH ≥ 4.5 mmol/L	Low‐GDP	1	.316	1	.685	1	.725
	High‐GDP	1.023 (0.979–1.069)		1.009 (0.965–1.055)		0.992 (0.946–1.039)	
BMI ≥ 24 kg/cm^2^	Low‐GDP	1	.001	1	< .001	1	< .001
	High‐GDP	0.925 (0.883–0.968)		0.884 (0.844–0.926)		0.840 (0.799–0.884)	
Abnormal metabolism state	Low‐GDP	1	< .001	1	< .001	1	< .001
	High‐GDP	0.562 (0.472–0.665)		0.552 (0.463–0.654)		0.533 (0.444–0.636)	

*Note*: Abnormal metabolic state: HbA1c ≥ 7%, blood pressure ≥ 130/80 mm Hg, TCH ≥4.5 mmol/L, or BMI ≥24 kg/m^2^. Model 1: unadjusted. Model 2: adjusted for sex and age. Model 3: sex, age, diabetes duration, family history of diabetes (yes or no), history of hypertension (yes or no), history of hyperlipidemia (yes or no), BMI (≥24 kg/m^2^ or < 24 kg/m^2^), physical activity (up to standard or not), alcohol consumption habits (drinking or not), and smoking habits (ideal smoking or not).

Abbreviations: BP, blood pressure; BMI, body mass index; CI, confidence interval; GDP, gross domestic product; OR, odds ratio; TCH, total cholesterol.

### 3.4 A healthy lifestyle had additive effects on achieving HbA1c control even among patients with higher GDP

**FIGURE 4:**
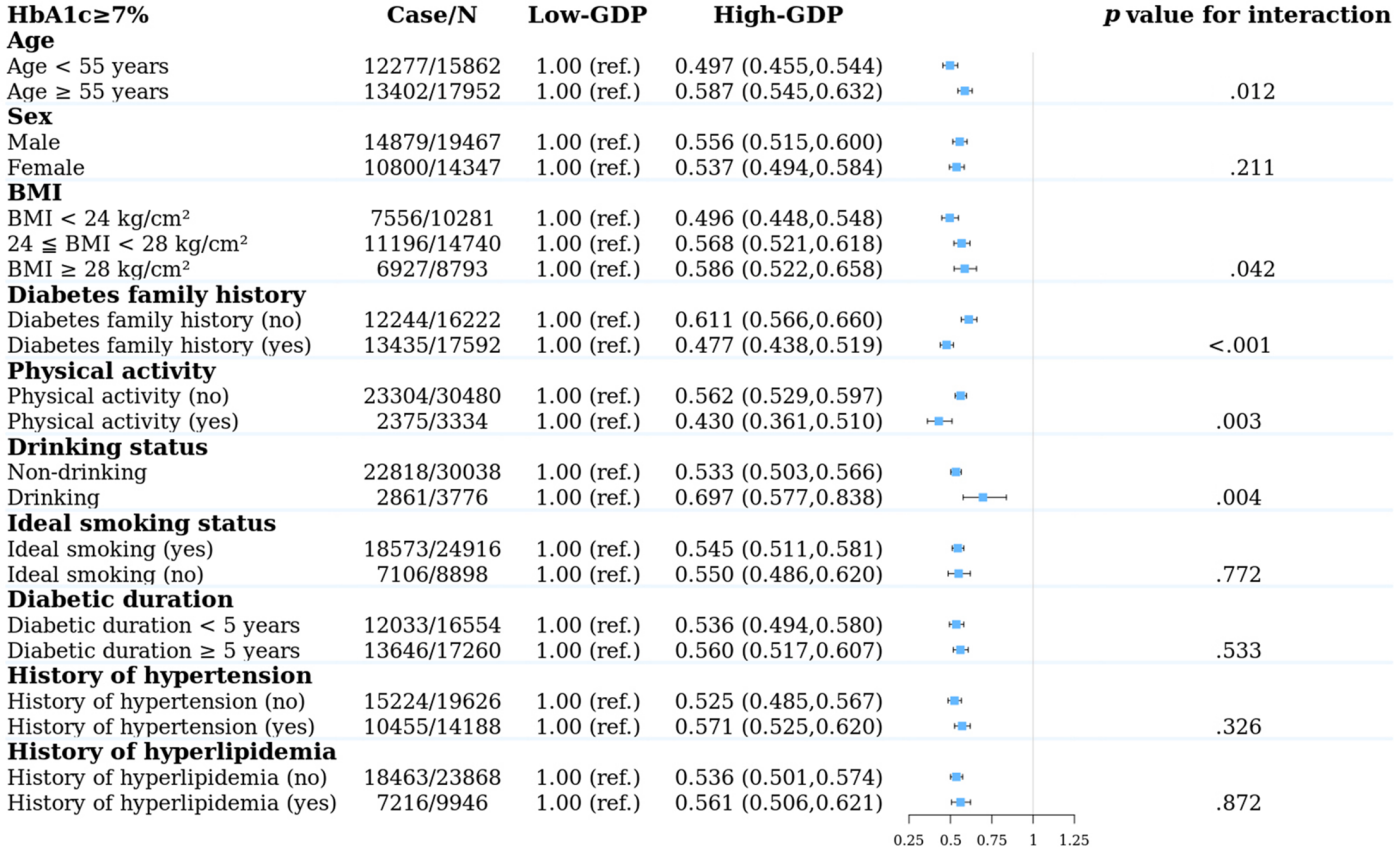
Stratified analysis of the proportion of glycated hemoglobin (HbA1c) ≥ 7% in different populations with different gross domestic product (GDP) per capita. *p* for interaction analysis was adjusted for the following potential confounders: sex, age, diabetes duration, family history of diabetes (yes or no), history of hypertension (yes or no), history of hyperlipidemia (yes or no), body mass index (BMI) (≥24 kg/m^2^ or < 24 kg/m^2^), physical activity (up to standard or not), alcohol consumption habits (drinking or not), and smoking habits (ideal smoking or not). When a specific indicator was used for subgroup population analysis, the indicator was not adjusted for.

We further explored the interaction between age, sex, BMI, diabetes family history, lifestyle (physical activity, alcohol consumption status, smoking status), medical history (history of hypertension and history of hyperlipidemia), and economic status with glycemic control (Figure 4). In the high‐GDP group, patients younger than 55 years old (OR = 0.497 vs. 0.587, *p* = .012), with a BMI < 24 kg/m^2^ (OR = 0.496 vs. 0.568 vs. 0.586, *p* = .042), and with a family history of diabetes (OR = 0.477 vs. 0.611, *p* < .001) had better HbA1c control. In addition, patients in the high‐GDP group who met the physical activity standard or were nondrinkers had better HbA1c control (OR for physical activities up to standard = 0.430 vs. 0.562, *p* = 0.003; OR for nondrinkers = 0.533 vs. 0.697, *p* = .004). However, there was consistently better glycemic control in the higher‐GDP group across other stratifications, such as sex, ideal smoking status, diabetic duration, history of hypertension, and history of hyperlipidemia. Therefore, in the high‐GDP group, participants with a younger age, a family history of diabetes, normal weight, and physical activity meeting the standard and who were nondrinkers were more likely to have better HbA1c control.

## 4 DISCUSSION

The subjects of our study were from 10 representative MMCs in regions with different economic levels in China. Our study included nearly all adults with T2DMs in 10 centers. To some extent, the patient population characteristics of the MMCs were close to the population characteristics of the area. Our results showed that BMI, BP, blood glucose, TGs, and the HOMA2‐IR of T2DM patients in the high‐GDP group were significantly lower than those in the low‐GDP group. Patients with lower GDP per capita had lower rates of HbA1c, BP, BMI, and comprehensive control. Furthermore, after adjusting for confounding factors, the risk of metabolic abnormalities was much lower in the high‐GDP group.

Our results were consistent with those of previous studies.^18^ A study from the capital Beijing showed that the control rates of blood glucose, BP, blood lipids, and weight were 30.9%, 30.2%, 17.4%, and 27.7%, respectively, among 917 diabetic patients. The comprehensive control rate of diabetes mellitus was only 2.0%, with slightly different diabetes comprehensive control goals as follows: FBG 4.4 ~ 7.0 mmol/L and HbA1c <7.0%, BP < 130/80 mm Hg, TC <4.5 mmol/L, male (female) HDL‐C > 1.0 (1.3) mmol/L, LDL – C < 2.6 (1.8) mmol/L, and BMI < 24.0 kg/m^2^.^19^ In the CCMR‐3B study, of the 25 454 patients, the patients with the highest household income were more likely to achieve BP < 130/80 mm Hg (adjusted OR = 1.16 [95% CI: 1.07–1.27]) but less likely to reach HbA1c < 7.0% (adjusted OR = 0.90 [95% CI: 0.83–0.98]) than those with the lowest income.^20^ Gomes et al found that very low economic status was independently associated with poor glycemic control in young Brazilian type 1 diabetes patients. Screening for diabetic complications and attaining glucose, lipid, and BP goals posed a challenge for these patients, and the low economic status of the majority of the patients may represent a barrier to reaching these goals.^21^ Compared with previous studies, our study sample size was larger, and the patients were from the 10 MMCs with the best quality control. Using GDP per capita as the economic evaluation could better explore the diabetes control rate in different economic regions to facilitate resource investment and management in policy‐making. The reasons for the poor metabolic control rate among people with a poor economic status may be attributable to health awareness, medical resources, and medical insurance. There was a significant correlation between GDP growth and medical economic investment.^22^ The increases in GDP per capita and population density promoted the improvement of public medical and health efficiency.^23^ Therefore, more attention and care should be offered to the lower economic regions by the government, medical organizations, and all sectors of society. With the MMC mission of one center, one stop, and one standard, a new metabolic disease management model was created based on standardized procedures and an internet health information platform. The analysis of these baseline data provided a practical basis to improve the diagnosis and treatment of diabetes in low‐GDP areas and achieve standardized treatment across the whole country.

For further interaction analysis of the proportion of HbA1c ≥ 7% in different populations, we found that younger patients and patients with a family history of diabetes were more likely to have better HbA1c control among patients in the higher GDP group. Although some previous studies have found that older diabetic patients had better glucose control than younger patients,^24^, ^25^ our study found that younger people with better economic conditions had better HbA1c control. This result was similar to that of Teufel.^26^ This may be related to the fact that young people currently pay increasing attention to their physical health. It was very interesting that we found that HbA1c control was better in patients with a family history of diabetes in the high‐GDP group. Patients with a parental history of T2DM were diagnosed at a younger age.^27^ David et al found that maternal family history was significantly associated with a lower risk of all‐cause mortality and cardiac mortality. Subjects with a family history may be more likely to recognize the risk factors and symptoms of diabetes and thus receive a diagnosis and start appropriate management (including that for no glycemic cardiovascular risk factors) at a relatively early stage.^28^ Therefore, patients with a family history of diabetes may care for their blood sugar to achieve better glycemic control.

We also observed a correlation between the HbA1c control rate and lifestyle behaviors among T2DM patients. We found that nonoverweight patients, individuals whose physical activity level was up to standard and nondrinkers were more likely to have controlled HbA1c levels among patients with higher GDP. Previous studies have shown the beneficial effect of a healthy lifestyle on glucose metabolism. A higher risk of diabetes was observed at a BMI of 23 kg/m^2^ or higher among 685 and 616 adults in low‐income and middle‐income countries, respectively, with a 43% greater risk of diabetes for men and a 41% greater risk for women compared with a BMI of 18.5–22.9 kg/m^2^.^25^ In middle‐income countries, urban percentage, agglomeration index, and GDP were significantly associated with T2DM prevalence, whereas in high‐income countries, physical inactivity and obesity were the main determinants of T2DM prevalence.^29^ It has also been found that patients with BMI less than 24 kg/m^2^ had better HbA1c control than overweight or obese patients. A survey follow‐up study from 1994 to 1997 among Kaiser Permanente Northern California members showed that alcohol consumption was linearly (*p* < .001) and inversely (*p* = .001) associated with HbA1c among diabetes patients.^30^ HbA1c (adjusted for age, sex, duration of diabetes, therapy) levels among patients with lower alcohol consumption levels were significantly lower than those among patients who drank alcohol.^31^ Lifestyle management, such as a balanced diet, regular exercise, and normal weight maintenance, is essential for the improvement of insulin resistance and good blood glucose control.^32^, ^33^ Therefore, healthy lifestyle behaviors had additive effects on achieving glycemic goals, even in individuals with better economic status.

Although the results of the present study were very interesting, our study had several limitations. Our study was a cross‐sectional study, although the number of participants was 37 454 and covered several cities. The subjects were adult T2DM patients who were willing to accept MMC management and follow‐up. But this was a nonrandom selection. There were likely to be some selection biases. The results would be more convincing if drug information had been provided. Another limitation was unmeasured confounders related to GDP levels, such as unemployment rate, medical insurance, medical resources, screening systems, access to specialists, job status, and educational levels.

In summary, our study suggested that T2DM patients with better economic conditions had a better metabolic control rate. Moreover, younger patients, those with a family history of diabetes, nonoverweight patients, individuals with physical activity levels up to standard, and nondrinkers were more likely to achieve glycemic control among individuals with better economic status. We should strengthen comprehensive DM management and encourage a healthy lifestyle in less economically developed regions in China. At the same time, we also provided real‐world evidence of strategies for precise diabetes management programs in different economic regions.

## CONFLICT OF INTEREST

The authors declare no competing financial interests.

## DISCLOSURES

The authors have no disclosures relevant to the present study. the National Metabolic Management Center in China, with advanced medical equipment and Internet of Things technology, was founded in 2016. The study protocol was approved by the Ethics Review Committee of Ruijin Hospital and the other participating centers. The study was performed in accordance with the Declaration of Helsinki. Comprehensive written informed consent was provided by all participants.

## Data Availability

The data sets generated during and/or analyzed during the current study are not publicly available but are available from the corresponding author on reasonable request.
